# Dietary Diversity and Child Development in the Far West of Nepal: A Cohort Study

**DOI:** 10.3390/nu11081799

**Published:** 2019-08-03

**Authors:** Andrew L. Thorne-Lyman, Merina Shrestha, Wafaie W. Fawzi, Monica Pasqualino, Tor A. Strand, Ingrid Kvestad, Mari Hysing, Neena Joshi, Mahendra Lohani, Laurie C. Miller

**Affiliations:** 1 Center for Human Nutrition, Department of International Health, Johns Hopkins Bloomberg School of Public Health, 615 N. Wolfe Street, Room E2545, Baltimore, MD 21205, USA; 2 Department of Child Health, Tribhuvan University Teaching Hospital, Kathmandu, Nepal; 3 Department of Global Health and Population, Harvard T.H. Chan School of Public Health, 665 Huntington Avenue, Building 1 Room 1108, Boston, MA 02115, USA; 4 Department of Nutrition, Harvard T.H. Chan School of Public Health, Boston, MA 02115, USA; 5 Department of Epidemiology, Harvard T.H. Chan School of Public Health, Boston, MA 02115, USA; 6 Division for Research, Innlandet Hospital Trust, Lillehammer, Norway and The Center for International Health, University of Bergen, Bergen, Norway; 7 Regional Center for Child and Youth Mental Health and Child Welfare, NORCE Norwegian Research Center, Bergen, Norway; 8 Department of Psychosocial Science, Faculty of Psychology, University of Bergen, Bergen, Norway; 9 Heifer International, Satobato Road, Hattiban, Kathmandu, Nepal; 10 Heifer International, 1 World Ave, Little Rock, AR 72202, USA; 11 Tufts University School of Medicine, 800 Washington St, Boston, MD 02111, USA

**Keywords:** dietary diversity, child development, animal source food, vegetable, fruit, milk, dairy, processed foods, stunting, growth

## Abstract

Poverty adversely affects child development through multiple pathways in low- and middle-income countries. Relationships between diet and child development are poorly understood. In this study, we aimed to explore these associations in a longitudinal cohort of 305 children in rural Nepal (baseline mean age 14 months), evaluating dietary diversity and the consumption of specific food groups at three timepoints over 1.5 years. Child development was assessed using the Ages and Stages questionnaire-version 3 (ASQ-3). Associations between the number of days that children consumed minimum dietary diversity (MDD) (≥4/8 items) and specific food groups over time (range 0–3) and total and subscale ASQ scores at age 23–38 months were estimated using multiple linear and logistic regression, dichotomizing scores at the lowest quartile. After adjusting for confounders, each additional day of consuming MDD was associated with a 35% reduction in the odds of low total ASQ score [OR 0.65, 95% CI (0.46, 0.92)]. The consumption of animal source foods [OR 0.64, (0.46, 0.89)], and vegetables/fruits [OR 0.60, (0.41, 0.90), but not processed foods [OR 0.99, (0.62, 1.59)] was associated with lower odds of low total development. Vegetables, fruits and animal source foods may be important for child development in this setting.

## 1. Introduction

It has been estimated that 250 million children under 5 years of age in low- and middle-income countries (LMICs) fail to reach their developmental potential [1]. Brain development begins soon after conception and progresses rapidly through sequential neurological processes that occur over time, building on one another [2]. Disruptions to these events can result in long-term consequences [2]. By limiting school readiness, academic performance, and economic opportunities, poor child development contributes to the intergenerational cycle of poverty [3].

Poverty increases exposure to psychosocial and biological risk factors that often occur simultaneously and compromise early brain development [4,5]. Strong links have been demonstrated between poverty and cognitive and language development [6,7,8]. These delays may be mediated through multiple mechanisms including increased stress, limited access to material and social resources, inadequate stimulation, and poor nutritional status [9,10,11,12].

Young children suffering from malnutrition may have poorer school achievement and cognitive and language abilities [2,13,14,15]. Although many nutrients are necessary for brain development, the impact of nutrient deficiencies depends on the timing and degree of nutrient deprivation, the possibility of recovery, and the child’s environment [16]. Exposure to infectious diseases, environmental toxins, and violence, all of which may coexist with nutrient deprivation in low- and middle-income countries, may also negatively impact a child’s developmental potential [4,17,18]. Psychosocial stimulation through early learning opportunities and responsive caregiver–child interactions, however, can enhance development even for children in difficult circumstances [5,19].

Due to the lack of available data on child development in LMICs, child stunting has often been used as a proxy for estimating the burden of poor child development. However, while stunting may be a useful marker of deficient environments, recent papers have argued that claims suggesting direct causal associations between stunting and child development are not grounded in evidence, in turn raising questions about what common causes give rise to both conditions [20,21]. A number of studies have shown that increased dietary diversity is associated with child height, albeit weakly [22,23,24]. However, little research has been undertaken in LMICs to examine whether dietary diversity in early childhood is associated with child development.

Dietary diversity indicators are relatively simple to measure and some have been validated against nutrient adequacy and other indicators [25,26,27]. Populations in low resource settings typically have cereal-based diets with few or no animal source foods (ASF) and few seasonal fruits and vegetables [28]. Poor dietary diversity is especially concerning for infants and young children, given limited gastric capacity and high nutrient requirements needed to support the rapid rate of growth and development of early life [28,29]. ASFs, including meat, fish, milk and eggs, are dense sources of nutrients that are commonly deficient in the diets of children in LMICs and difficult to obtain in adequate quantities from plant source foods alone [30,31,32]. Studies conducted in LMICs have found the intake of ASFs to be positively associated with dietary quality as well as cognitive development among children [30,33,34]. In comparison, relationships between child consumption of vegetables and fruits or processed foods and child development have not been well studied in LMICS. 

Few studies in rural areas of LMICs have explored the predictors of child development [35,36]. One area in need of more study is the influence of dietary quality in early childhood on child development indicators in low-resource rural settings. The present study addressed this question through a longitudinal cohort study. The study was nested within a randomized trial of a livestock intervention by Heifer International in Banke, Nepal. The current study was conducted among young children whose families participated in this trial. The objectives of the current study were to (1) explore the association between dietary diversity, animal source food consumption, vegetable and fruit consumption, and processed food consumption during early childhood and overall child development and (2) examine the associations between each of these exposures and specific domains of child development.

## 2. Materials and Methods 

This longitudinal cohort study was nested within a 48-month randomized longitudinal-control impact evaluation [37] (ClinicalTrials.gov identifier NCT03516396) investigating the importance of various components of a livestock-based multisectoral community development intervention on growth and diet outcomes of children. The setting for the present study was in Banke district, a remote rural area located in far Western Nepal, populated mostly by low-income subsistence farmers. Heifer International, Nepal, maintained a list of communities in the area interested in participating in community development activities. Three non-adjacent communities from the same agro-ecological zone were identified based on their similar sociodemographic characteristics. The communities were randomly assigned to one of three arms: (1) a full intervention package consisting of community development, livestock training, and a nutrition curriculum (“Full Package”), (2) livestock training and nutrition curriculum only (“Partial Package”), and (3) the “Control group”, that received no inputs. The “Partial Package” and Control group communities received the Heifer full intervention after the research period of 24 months ended.

Families were identified within each Village Development Committee (an administrative unit in Nepal) and invited to participate in the study. Children with physical or neurologic handicaps that prevented ingestion of a normal diet for age or children with severe inter-current illnesses at the time of survey were excluded. Informed verbal consent was obtained prior to enrollment due to high illiteracy in the population, and at each contact, participants were reminded that they could withdraw from the study, discontinue the study visit, or decline to answer any of or all the questions at any time. Once enrolled, the participants were visited three times: a baseline assessment (Round 1) was conducted in August 2013, a follow-up visit (Round 2) in May 2014, and another follow-up visit (Round 3) in December 2014, forming a total follow-up period of approximately 16 months. At all three visits, data was collected on diet, anthropometry, animal ownership and other variables.

During the Round 3 visit, all children aged 23–38 months from across the intervention groups were invited to participate in the sub-study in which a child development screening tool, the Ages and Stages Questionnaire-3 “home procedure”, ASQ-3, was performed, and this sub-population forms the basis for this paper. This specific age group was chosen to simplify the training of enumerators and administration of the ASQ-3 test in the field.

Data collection was performed by a local field research NGO (Valley Research Group) not connected to *Heifer*. Field supervisors monitored the performance and activities of the Field Enumerators and conducted daily reviews of the data collection to allow rapid identification and correction of errors and omissions. Enumerators were trained at the beginning of the project with 7 days of orientation to the project, field practice testing in two villages not included in the project sites, and ongoing quality control activities to monitor and maintain inter-observer reliability. A five-day refresher training, including field practice, was conducted prior to each additional field visit. An additional five days of training in administration of developmental testing, including practical training, was provided prior to the Round 3 visit. Field enumerators traveled in pairs to conduct the visits, during which a 145-item questionnaire was completed with the female head of household or her designee. A supervisor was also present for part of each visit. An additional supervisor, highly experienced in administration of the ASQ-3, traveled with the team and closely supervised their activities for the first 2 weeks of the 4-week period of data collection.

Consumption in the past 24 h of 16 specific foods/food groups was ascertained for all children in the household aged 1–60 months at each survey [38]. The foods were aggregated into 8 dichotomous food group categories, representing yes/no consumption by the child over the past 24 h to create an Individual Dietary Diversity Score (IDDS): starchy staples (grains and white potatoes), pro vitamin-A rich fruits and vegetables, other fruits and vegetables, flesh foods (offal, meat, and fish), eggs, legumes, nuts and seeds, milk and dairy products, and oils [39]. Dichotomous variables were created for each data collection round, with diverse diets defined as those representing a consumption ≥4 times and non-diverse diets defined as consumption less than this amount. In addition, dichotomous variables were created for each of the three data collection rounds, representing any consumption of animal source foods (mean, offal, fish, eggs, milk or dairy productions), “vegetables” (green leafy vegetables, provitamin A rich fruits or vegetables or other fruits and vegetables, which, in Nepal, consists mostly of vegetables), and processed foods (biscuits, noodles). To characterize the diet over the period of young childhood, the number of days that children met minimum dietary diversity or had consumed any item in each of these food groups over the three data collection rounds was summed, resulting in a maximum of 3 days and a minimum of 0.

The Ages and Stages Questionnaire-3 (ASQ-3) is a 30-item tool that evaluates five different subscales of child development [40,41]. The questionnaire generates an overall measure of child development as well as separate scores in gross and fine motor, personal-social, problem-solving, and communication areas. The standardized screening instrument has been used to assess child development in many global contexts including in peri-urban Nepal and India [40,42,43,44]. The age-specific questionnaires corresponding to the following age groups were used in this study: 23–26, 27–29, 30–32, 33–35 and 36–38 months. The questionnaires were translated into Nepali and translated back into English by an English professor at Tribhuvan University. The back translations were discussed with the child psychologists on our team and used to finalize the Nepali version of the questionnaire. The questionnaire was pilot tested in a village unconnected to and distant from the research site. Local adaptations were made for certain items when those items were found to not apply to the local context.

The ASQ-3 questionnaire contains six items for each of the five subscales for a total of 30 items. The total possible score range for each subscale is 0–60 (resulting in a total possible score range of 0 to 300). One advantage of the ASQ-3 is that it can either be assessed directly by trained examiners or answered by caregivers [43]. In this study, we used both approaches, prioritizing observations by trained examiners over caregiver responses, with the proportion of items observed vs. responded by caregivers presented in Figure S1. The examiners used a standardized toolkit consisting of tools used to elicit responses to questions (e.g., blocks, crayons, paper, balls, scissors) [42]. Following data collection, we removed scale items with no variability and reweighted the remaining items of each subscale such that each item contributed.

The Pearson correlation coefficients between each of the subscales and the total ASQ-3 score are presented in Table S1. Most of the correlations between subscales were moderate, and those between subscales and the total score were strong, and all were found to be highly significant (p < 0.001). The internal consistency of each of the subscales and the total scale was assessed for each age group using standardized Cronbach alphas (Table S2). Of the 25 age group subscales (5 scales for each of the 5 age groups tested), 13 were classified as moderately consistent (0.4 to 0.6), 8 were satisfactory (0.6 to 0.8), and the remaining 4 showed weak consistency (below 0.4). As no validated/standardized threshold exists to characterize poor development using the ASQ in Nepal, we defined children in the bottom 25% of the ASQ-3 total score or each of the subscales as having poorer development outcomes, as has been done in other studies in the context of Nepal [42,45].

Basic information about demographics and socioeconomic status was collected from each household. Child age was determined by inspection of the birth or the vaccine certificate. A wealth index was constructed using principal components analysis based on assets and housing construction materials, similar to the approach used in the Demographic and Health Surveys [46]. Animal ownership, annual income per household and per capita, and amount of land owned were also collected as additional indicators of household wealth. The number of animals owned was converted to a standardized score using FAO Global Livestock Units [47]. Respondents were asked about the educational attainment of the highest educated male and the mother of the child with 6 categories and compressed into 3 categories.

All statistical analyses were conducted in SAS 9.4. Unadjusted and multivariable linear regression analysis was conducted, treating total child development as a continuous outcome and dietary exposures. Odds ratios and 95% confidence intervals were also calculated for unadjusted and multivariable adjusted logistic regression models examining low total development scores (lowest 25%) and low development scores for each subscale (below the age-specific cutoff). Adjusted models included a priori variables suspected to confound the relationship between diet and child development, including child age, maternal education, and wealth quintiles, and analyses were also adjusted for randomized trial assignment.

This study was approved by the Nepal Health Research Council (Ref. No. 1369, 1327) and the Tufts University Institutional Review Board.

## 3. Results

Of the 960 households and 1333 children <60 months of age who were enrolled and randomized in the original trial, 319 children between the ages of 23 and 38 months at the time of the third household survey were eligible for the developmental (ASQ) assessment (Figure S2). Ten mothers could not be contacted, two of the eligible children had died, leaving 307 children that completed developmental testing in Round 3 of data collection. We excluded two children with total development scores <50 from analyses, as we felt these scores were implausible, leaving 305 children. The characteristics of the children and their households are shown in Table 1. Similar proportions of children came from the three randomization areas, although the proportion of those in the Heifer full package intervention area was lower than the other two groups.

### 3.1. Diet Characteristics

Over half of children met at least the threshold for minimum dietary diversity at each individual round of data collection, but only about a quarter met it for all three rounds of data collection (Table 2). Between 40–60% of children consumed at least one animal source food at each data collection round, with about 18% of children not consuming any animal source foods across rounds, about 40% of children consuming animal source foods 2 of 3 days, and 16% consuming on all three days. Examining the distribution of 505 specific ASFs consumed over time, milk was the most consumed (57.6% of all reported consumption episodes of ASFs), followed by meat (30.1%), fish (8.3%) and offal (3.9%). Stark seasonal variations in vegetable/fruit consumption were evident with nearly 90% of children consuming these items at round 3 (post-harvest) vs. only 36–50% in previous rounds (pre-harvest). Processed food consumption was similar at each individual round and about two thirds of children had consumed processed foods in the past 24 h at all three data collection rounds.

### 3.2. Diet and developmental scores

The total ASQ-3 scores ranged from 82 to 300, with a median of 218 and an inter-quartile range (IQR) of 183 to 250 (Table 3). The median of each subscale ranged from 40 to 60, and the Gross Motor subscale had the highest median score. In multivariable-adjusted logistic regression models, each additional day that children met or exceeded the dietary diversity threshold of at least 4 food groups was associated with 33% lower odds of being in the bottom 25th percentile of total ASQ score (OR 0.65, [95% CI 0.46, 0.92]) (Table 4). However, in linear regression models, although we observed a significant 9.6 point [95% CI 3.5, 15.6] increase in total ASQ score with each additional day of dietary diversity in unadjusted analyses, adjusting for confounders attenuated the relationship to a 4.6 [95% CI −2.0, 11.2] point increase. In logistic models, while the magnitude of adjusted OR coefficients (OR < 0.75) suggested possible protective associations between greater dietary diversity and the odds of having low scores, none of the scores were statistically significant after adjustment for confounders.

The continuous association between the number of days that animal source foods were consumed and total ASQ suggested a marginally significant 6.1 [95% CI 0.2, 12.1] point greater score associated with each additional day of consumption of ASFs after adjusting for confounders (Table 5). Animal source food consumption was also associated with a significant 36% lower odds of having a total ASQ-3 score (OR 0.64, [95% CI 0.46, 0.89]) and 32% lower odds of having a low communication score (OR 0.68, [95% CI 0.50, 0.94]). Examining associations between specific food items and development scores (Table S3) revealed similar associations for both dairy 6.0 (−0.3, 12.3) and eggs 5.5 (−3.4, 14.3) in adjusted linear models, although these were not statistically significant. Dairy consumption was associated with 42% lower odds of low total development score (OR 0.58 [95% CI 0.39, 0.85] as well as 33% lower odds of a low communication score (OR 0.66 [95% CI 0.46, 0.95)] in multivariable adjusted models (Table S3).

Each additional day of consumption of vegetables was associated with a statistically significant 9.3 point [95% CI 2.4, 16.3] higher total score on the total ASQ-3 in adjusted linear regression models, and a 40% lower risk of falling in the lowest 25% of the total ASQ-3 distribution (OR 0.60 [95% CI 0.41, 0.90). More frequent vegetable consumption was associated with protective odds of a low score for three subscales (communication, fine motor, and personal-social) and a marginally significant association was observed for the problem-solving subscale after adjusting for confounders (Table 5). Specifically, green leafy vegetable consumption was associated with ~40% lower odds of a low score on the fine motor, problem solving and personal-social subscales after confounder adjustment (Table S3).

Processed food consumption was not associated with total development scores in linear or logistic models, and findings also suggested null relationships with communication and gross motor subscales. The ORs of both the fine motor and the problem-solving subscales suggested the possibility of increased odds of poor development associated with an increased consumption of processed foods, although the confidence bounds of these estimates also included the null. Increased processed food consumption was associated with a trend towards reduced odds low personal-social scores.

## 4. Discussion

In this longitudinal cohort study conducted in rural Nepal, we examined children’s diet in relation to their developmental performance. We found that dietary diversity over a child’s early life was associated with a better overall developmental performance after adjusting for potential confounders. Children who consumed greater amounts of ASF in early childhood performed better on developmental testing scores, a finding consistent with previous observational and experimental studies [33,48,49]. Interestingly, we also found that vegetable and fruit consumption was also associated with better development. These findings suggest the importance of early dietary diversity during the complementary feeding period as a potential influence on child development, as one dimension of a cluster of poverty-related factors that likely influence the trajectory of child development.

Dietary diversity has been validated as an indicator of dietary quality for infants and young children in low- and middle-income countries [27]. A number of studies have also shown that greater dietary diversity was associated with positive child growth outcomes [28]. However, few studies have explored associations between children’s diets and development outcomes in such settings. One multi-cohort study found dietary diversity to be a consistent predictor of language development among children at 18 months of age in three of four prospective study cohorts in Ghana, Malawi, and Burkina Faso [36]. This finding is also consistent with our observations related to both ASF and vegetable/fruit consumption and better scores on the communication subscale. A study in Bihar, India found that dietary diversity was correlated with motor and mental development, and that stimulation, gross motor development, and fine motor development were significant mediators in the relation between dietary diversity and mental development among children 6 to 18 months of age [50].

In low-resource settings, children’s diets are often cereal-based with few or no ASFs and few vegetables and fruits. It is therefore challenging for these children to attain a high dietary diversity [25,28]. Low consumption of ASF among children is a particular problem, as these foods are dense sources of energy and nutrients that play essential roles in growth and development [31]. Evidence indicates that diets deficient in ASF are associated with poorer cognitive performance among children [31]. The Nutrition Collaborate Research Support Program found that children of various ages who consumed ASF performed better on cognitive tests than those who did not [33,51]. ASFs are recognized for their role in cognitive development as they are rich in protein, energy, omega-3 fatty acids, iron, zinc, vitamin A, vitamin B12, and vitamin D [30,31,52]. The findings of a study in peri-urban Nepal suggest that early vitamin B12 deficiency is a possible cause of adverse developmental outcomes, emphasizing the potential need for ASFs in the diets of children [42]. In line with the current findings, general development and problem solving were the domains that were related to early vitamin B12 status. Similarly, in an intervention trial in North Indian urban children, children who received vitamin B12 and folic acid had higher total, gross motor and problem-solving skills on the ASQ-3 compared with the placebo group [53]. Another intervention trial showed that general problem-solving was associated with an intervention providing meat to school children [33]. Additionally, micronutrients in ASFs tend to have greater bioavailability than micronutrients from plant foods [32,54]. It should be noted that most previous studies exploring the relationship between the consumption of ASFs and child growth and development focused on milk, meat, or eggs as food supplements, but did not investigate the impact of usual diets on cognitive performance, as was done in this study [33,51,55].

The current study also found an association between vegetable/fruit consumption and better development scores. Provitamin A-rich vegetables and fruit, which are often lacking in the diets of poorer populations, contribute to the recommended dietary intake of vitamin A and other micronutrients, and vegetables and fruit rich in ascorbic acid act as enhancers of non-heme iron and folate absorption [28,56,57]. Vegetables and fruits are rich in nutrients needed for brain development such as folate, vitamin A and iron [16]. It is plausible that intake of these nutrients through increased vegetable consumption would be beneficial for development. Through our search of the literature, we did not find any other studies assessing the association between vegetable intake and child development in low- and middle-income countries. In one study conducted in Finland, vegetable consumption was associated with greater cognition scores in boys, but not girls [58]. One study conducted in Bangladesh, India, and Nepal found that a lower intake of vegetables and fruit was associated with higher odds of depression, suggesting that diets low in these food groups may have a negative impact on mental health outcomes [59]. However, the current study appears to be the first—that we are aware of—to find a relationship between vegetable intake and child cognition in a low-income setting.

In Nepal and many other countries worldwide, efforts are underway to diversify food production beyond the traditional focus on rice and other staple foods through efforts that include livestock interventions and homestead gardening. Dietary diversity is often considered a proximate outcome of such efforts, with reduction in stunting (a proxy for child development) often framed as the ultimate goal. Our findings suggest that it may be possible to directly measure various aspects of child development using field-friendly assessment tools, such as the ASQ-3, as an additional measure of the effectiveness of such programs. However, it is important to emphasize that the expected effects of programs on child development would likely be greater where agricultural or livelihood interventions are coupled with early childhood development interventions.

The positive association between dietary quality and child development persisted even after adjusting for socioeconomic status. There is strong evidence that socioeconomic factors influence child growth and development. Poverty has been found to increase the risk factors for poor development and to limit protective factors and opportunities for stimulation in the environment [2,4,60,61,62]. A cohort study conducted under MAL-ED in Tanzania, for example, determined that household socioeconomic status was the strongest factor associated with child development at 15 months of age [6]. Lower socioeconomic status is known to be associated with a lower nutritional status, which can also contribute to poor development. The current study adds to the literature on the detrimental effects of poverty by suggesting potential mechanisms that occur between socioeconomic status and child development through nutritional adequacy and intake of certain nutrient-dense food groups.

Maternal education, which is among the risk factors for poor development, was also found to be associated with child development outcomes in the current study. In an analysis of the survey data from five countries in South Asia including Nepal, a lack of maternal education was among the strongest correlates of poor child nutritional status [62]. Studies in developed countries have also found a positive relationship between a mother’s level of education and her child’s behavior and school performance [61,63]. The results from this study therefore support the evidence for integrated interventions that aim to offset the negative effect of poverty and low maternal education, which may exist together in populations suffering from undernutrition, on child nutritional status and cognitive development [16].

A recently published cross-sectional study in the Kathmandu Valley found that unhealthy snack foods and beverages made up an average of 24.5% of the total energy intake of children 12–23 months of age, that consumption is associated with lower nutrient adequacy, and that greater consumption was inversely associated with length-for-age z-score [64]. That study highlighted the need for further research on the role of such foods in young children’s diets and for more research on the nutritional and functional outcomes associated with consumption. The findings of the present study suggest that processed snack food consumption by young children extends to even more remote areas of Nepal and, unlike the consumption of healthier foods, was not associated with higher child development scores.

Our study had a number of important strengths, including a longitudinal design, its potential generalizability to many similar rural village settings in Nepal, and its focus on an age group that is normally the period in which growth faltering occurs. Another strength was that multiple dietary measures collected over time reduced exposure misclassification compared with one-time measures [24]. Study limitations included the relatively small sample size, which may have resulted in imprecise effect estimates. Another limitation was that the ASQ-3 tool has not been validated against a gold standard measurement in the context of Nepal and that there are accordingly no local norms. However, the tool has been used successfully in other contexts in Nepal and other South Asian settings [42,43,44]. As an observational study, we cannot rule out the possibility that unmeasured confounding could have influenced our results, and we did observe some attenuation after adjusting for measured confounders. Future efforts should explore additional dimensions of socioeconomic status not captured by our wealth score such as parental care, time availability, the home environment, and parental depression, some of which might be correlated with feeding practices as well as child development indicators. Additionally, our dietary measurement was fairly imprecise, and did not account for quantity, which may be particularly important for animal source foods, given the relatively high concentrations of certain nutrients.

In conclusion, our study built upon the evidence that dietary diversity in early childhood is associated with better growth, suggesting that it is also associated with improved overall child development scores in rural Nepal. Given the expanding consumption of ultra-processed foods, it may be important to continue to promote the consumption of vegetables, fruits, and animal source foods by young children. As the evidence base associating diet and child growth as well as development remains largely based on observational studies, randomized trials exploring the effectiveness of interventions to diversify diets in low-income settings on child development may be needed to help inform programs and policies.

## Figures and Tables

**Table 1:** Demographic and socioeconomic characteristics of study participants at baseline.

	*n*	Mean (SD) or %
Child sex- female (%)	305	47.9
Child age at baseline (months)	303	14.9 (4.8)
Maternal education (%)		
None/non formal education	225	73.8
At least some primary	60	19.7
At least some secondary or SLC	20	6.6
Highest male education in household (%)		
None	113	37.3
At least some primary	97	32.0
At least some secondary or SLC	67	22.1
No male in household/female headed household	26	8.6
Land owned m^2^ (mean, sd)	305	8255 (14838)
Wealth score	306	0.12 (1.04)
Study area (%)		
Control	104	34.1
Intervention	84	27.5
Partial intervention	117	38.4
Height-for-age z score	299	−1.84 (1.56)
Weight-for-age z score	299	−1.87 (1.09)
Weight-for-height z score	299	−1.24 (1.07)
Head circumference z score	299	−1.17 (1.18)
Stunted HAZ < −2) (%)	299	44
Wasted (WHZ < −2) (%)	299	24
Underweight (WAZ < −2) (%)	299	46
Microcephalic (HCZ < −2) (%)	299	25

**Table 2:** Dietary diversity, animal source food consumption and any vegetable consumption in the previous 24 h by round and sum of rounds.

Individual Rounds of Data Collection	Dietary Diversity ≥ 4 Groups (%)	Any Animal Source Food Consumption (%)	Any Vegetable Consumption (%)	Any Processed Food Consumption (%)
Round 1 ^1^ (*n* = 299)	53.5	40.5	49.2	78.9
Round 2 ^1^ (*n* = 287)	62.4	59.6	36.6	87.8
Round 3 ^1^ (*n* = 305)	70.5	60.0	88.2	84.6
Sum of days consumed across rounds ^2^				
0 days	11.0	17.8	4.6	1.4
1 day	20.2	24.9	35.8	9.6
2 days	40.8	40.9	40.4	25.9
3 days	28.0	16.4	19.2	63.1
Total	100.0	100.0	100.0	100.0

^1^ Percent represents the proportion of children that reported consuming at least once in the previous day; ^2^ Percent represents the sum of days that children consumed each food or had a diverse diet over all three surveys.

**Table 3:** Distribution of ASQ-3 total and subscale scores (*n* = 305).

	Median	IQR
Personal social	40	30–50
Problem solving	40	30–50
Gross motor	60	40–60
Fine motor	40	30–50
Communications	50	40–60
Total	218	184–250

IQR, inter-quartile range.

**Table 4:** Unadjusted and adjusted relationships between number of days meeting minimum dietary diversity over the three visits in early childhood and ASQ-3 scores (total and by domain), *n* = 282.

Variables	# Days Meeting Minimum Dietary Diversity, Continuous	
	Value (95% CI)	*P*
**Linear regression**		
Total ASQ-3		
Crude β	9.6 (3.5, 15.6)	<0.0001
Adjusted β ^1^	4.6 (−2.0, 11.2)	0.17
**Logistic regression** ^2^		
Total ASQ-3		
Crude OR	0.61 (0.46, 0.81)	<0.001
Adjusted OR ^1^	0.65 (0.46, 0.92)	0.02
Communication		
Crude	0.72 (0.55, 0.94)	0.02
Adjusted ^1^	0.79 (0.57, 1.09)	0.15
Gross motor		
Crude	0.71 (0.54, 0.93)	0.02
Adjusted ^1^	1.02 (0.72, 1.46)	0.90
Fine motor		
Crude	0.97 (0.75, 1.25)	0.80
Adjusted ^1^	0.92 (0.67, 1.26)	0.60
Problem solving		
Crude	0.85 (0.66, 1.09)	0.20
Adjusted ^1^	0.92 (0.68, 1.23)	0.56
Personal-social		
Crude	0.72 (0.56, 0.94)	0.01
Adjusted ^1^	0.84 (0.60, 1.17)	0.31

^1^ Adjusted for maternal education (two categories), wealth quintile, child age, and randomized intervention group; ^2^ OR of scoring in the bottom 25% of the population distribution.

**Table 5:** Unadjusted and adjusted relationships between diet (number of days of animal source food consumption, vegetable consumption, and processed food consumption over 3 days in early childhood) and child development/low child development (lowest 25%), *n* = 282.

Variables	Animal Source Food, Each Day Consumed		Any Vegetable, Each Day Consumed		Processed Food, Each Day Consumed	
	Value (95% CI)	*P*	Value (95% CI)	*P*	Value (95% CI)	*P*
**Linear regression**						
Total ASQ-3 ^2^						
Crude β	9.6 (3.9, 15.4)	<0.01	15.7 (9.0, 22.3)	<0.0001	−3.9 (−11.6, 3.9)	0.32
Adjusted β ^1^	6.1 (0.2, 12.1)	0.04	9.3 (2.4, 16.3)	<0.01	−2.6 (−11.1, 6.0)	0.55
Logistic regression						
Total ASQ-3 ^2^						
Crude OR	0.60 (0.45, 0.80)	<0.001	0.51 (0.36, 0.73)	<0.001	1.04 (0.71, 1.51)	0.85
Adjusted OR ^1^	0.64 (0.46, 0.89)	<0.01	0.60 (0.41, 0.90)	0.01	0.99 (0.62, 1.59)	0.97
Communication						
Crude	0.63 (0.48, 0.84)	<0.01	0.59 (0.42, 0.83)	<0.01	1.19 (0.82, 1.74)	0.36
Adjusted ^1^	0.68 (0.50, 0.94)	0.02	0.69 (0.47, 1.00)	<0.05	1.20 (0.76, 1.91)	0.44
Gross motor						
Crude	0.87 (0.66, 1.14)	0.31	0.67 (0.49, 0.94)	0.02	0.93 (0.66, 1.33)	0.71
Adjusted ^1^	1.10 (0.79, 1.54)	0.57	1.07 (0.71, 1.62)	0.76	1.04 (0.64, 1.70)	0.88
Fine motor						
Crude	0.94 (0.73, 1.22)	0.66	0.65 (0.47, 0.88)	<0.01	1.51 (1.04, 2.19)	0.03
Adjusted ^1^	1.00 (0.74, 1.34)	0.98	0.60 (0.42, 0.86)	<0.01	1.22 (0.79, 1.91)	0.37
Problem solving						
Crude	0.77 (0.60, 0.99)	<0.05	0.70 (0.52, 0.94)	0.02	1.31 (0.93, 1.85)	0.12
Adjusted ^1^	0.84 (0.64, 1.12)	0.23	0.76 (0.54, 1.05)	0.09	1.41 (0.93, 2.14)	0.11
Personal-social						
Crude	0.87 (0.67, 1.12)	0.28	0.61 (0.45, 0.84)	<0.01	0.81 (0.58, 1.13)	0.22
Adjusted ^1^	0.96 (0.71, 1.31)	0.82	0.78 (0.54, 1.13)	0.02	0.65 (0.41, 1.03)	0.07

^1^ Adjusted for maternal education (2 categories), wealth quintile, child age, and randomized intervention group; ^2^ OR of scoring in the bottom 25% of the population distribution.

## Supplementary Materials

The following are available online at https://www.mdpi.com/2072-6643/11/8/1799/s1, Figure S1. Percentage of items in each subscale observed by examiners during sessions, Figure S2. Study flow, Table S1. Standardized alphas by total ASQ-3 scale and subscales, Table S2. Pearson product moment correlation coefficients between the subscales and the total ASQ-3 score (all ages), Table S3. Unadjusted and adjusted relationships between specific food items and child development/low child development (lowest 25%), *n* = 282.
