# Supplementary material for: Dietary Diversity and Child Development in the Far West of Nepal: A Cohort Study
Source: Nutrients. 2019 Aug 3;11(8):1799. doi: 10.3390/nu11081799 (PMC6722734; doi:10.3390/nu11081799)
Supplement: Supplementary file 1 [file nutrients-11-01799-s001.pdf]

Supplementary materials

Table S1. Standardized alphas by total ASQ-3 scale and subscales[1]

Age Interval	N	Communication Std. α	Gross Motor Std α	Fine motor Std. α	Problem solving Std. α	Personal social Std. α	Total
35-39 months	83	0.19	0.67	0.72	0.42	0.46	0.67
32-34 months	62	0.51	0.43	0.72	0.58	0.58	0.82
29-31 months	44	0.75	0.65	0.61	0.68	0.51	0.73
25-28 months	64	0.58	0.66	0.40	0.58	0.27	0.74
23-25 months	54	0.56	0.54	0.29	0.39	0.49	0.75

ASQ-3 : Ages and Stages Questionnaire, 3rd Edition, [1]Interpretation: alpha >0.80-highly, 0.6-0.8-satisfactory, 0.4 to 0.6 moderately consistent

Table S2. Pearson product moment correlation coefficients between the subscales and the total ASQ-3 score (all ages)[1,2]

	Communication	Gross motor	Fine motor	Problem solving	Personal social
Communication					
Gross motor	0.30				
Fine motor	0.33	0.32			
Problem solving	0.41	0.23	0.40		
Personal social	0.35	0.43	0.31	0.41	
Total	0.68	0.65	0.69	0.70	0.74

ASQ-3 : Ages and Stages Questionnaire, 3rd Edition

[1]All correlations are significant, $p<0.001$; [2] Interpretation: 0.5 to 1.0=strong, 0.3 to 0.5=moderate, 0 to 0.3=weak

Table S3. Unadjusted and adjusted relationships between specific food items and child development/ low child development (lowest 25%), n=282

Variables	0 days consumed	Green leafy vegetables, each day consumed		Eggs, each day consumed		Dairy, each day consumed	
		Value (95% C I)	*P*	Value (95% C I)	*P*	Value (95% C I)	*P*
**Linear regression**							
Total ASQ-3[2]							
Crude β	ref	13.8 (6.1, 21.4)	<0.001	4.1 (-5.0, 13.2)	0.38	11.8 (6.0, 17.5)	<0.0001
Adjusted β[1]	ref	11.7 (4.1, 19.4)	<0.01	5.5 (-3.4, 14.3)	0.22	6.0 (-0.3, 12.3)	0.06
**Logistic regression**							
Total ASQ-3[2]							
Crude OR	ref	0.54 (0.36, 0.81)	<0.01	0.86 (0.55, 1.35)	0.51	0.51 (0.36, 0.71)	<0.0001
Adjusted OR[1]	ref	0.54 (0.34, 0.86)	<0.01	0.87 (0.52, 1.45)	0.59	0.58 (0.39, 0.85)	<0.01
Communication							
Crude	ref	0.69 (0.47, 1.02)	0.06	0.81 (0.52, 1.27)	0.36	0.58 (0.42, 0.79)	<0.001
Adjusted[1]	ref	0.74 (0.49, 1.13)	0.16	0.78 (0.48, 1.28)	0.33	0.66 (0.46, 0.95	0.02
Gross motor							
Crude	ref	0.65 (0.44, 0.95)	<0.01	1.15 (0.76, 1.73)	0.51	0.69 (0.51, 0.93)	0.12
Adjusted[1]	ref	0.85 (0.54, 1.34)	0.48	1.12 (0.68, 1.85))	0.65	1.03 (0.72, 1.47)	0.89
Fine motor							
Crude	ref	0.71 (0.50, 1.00)	0.052	1.20 (0.81, 1.77)	0.37	0.89 (0.68, 1.15)	0.36
Adjusted[1]	ref	0.62 (0.42, 0.93)	0.02	1.21 (0.79, 1.86)	0.39	1.04 (0.76, 1.43)	0.81
Problem solving							
Crude	ref	0.66 (0.47, 0.93)	0.02	0.84 (0.56, 1.24)	0.37	0.73 (0.56, 0.94)	0.02
Adjusted[1]	ref	0.64 (0.44, 0.93)	0.02	0.87 (0.57, 1.33)	0.51	0.79 (0.58, 1.06)	0.12
Personal-social							
Crude	ref	0.71 (0.50, 1.00)	0.052	0.82 (0.55, 1.24)	0.35	0.90 (0.70, 1.17)	0.43
Adjusted[1]	ref	0.59 (0.38, 0.91)	0.02	0.88 (0.55, 1.41)	0.58	0.96 (0.69, 1.33)	0.81

[1]Adjusted for maternal education (2 categories), wealth quintile, child age, and randomized intervention group [2]OR of scoring in the bottom 25% of the population distribution

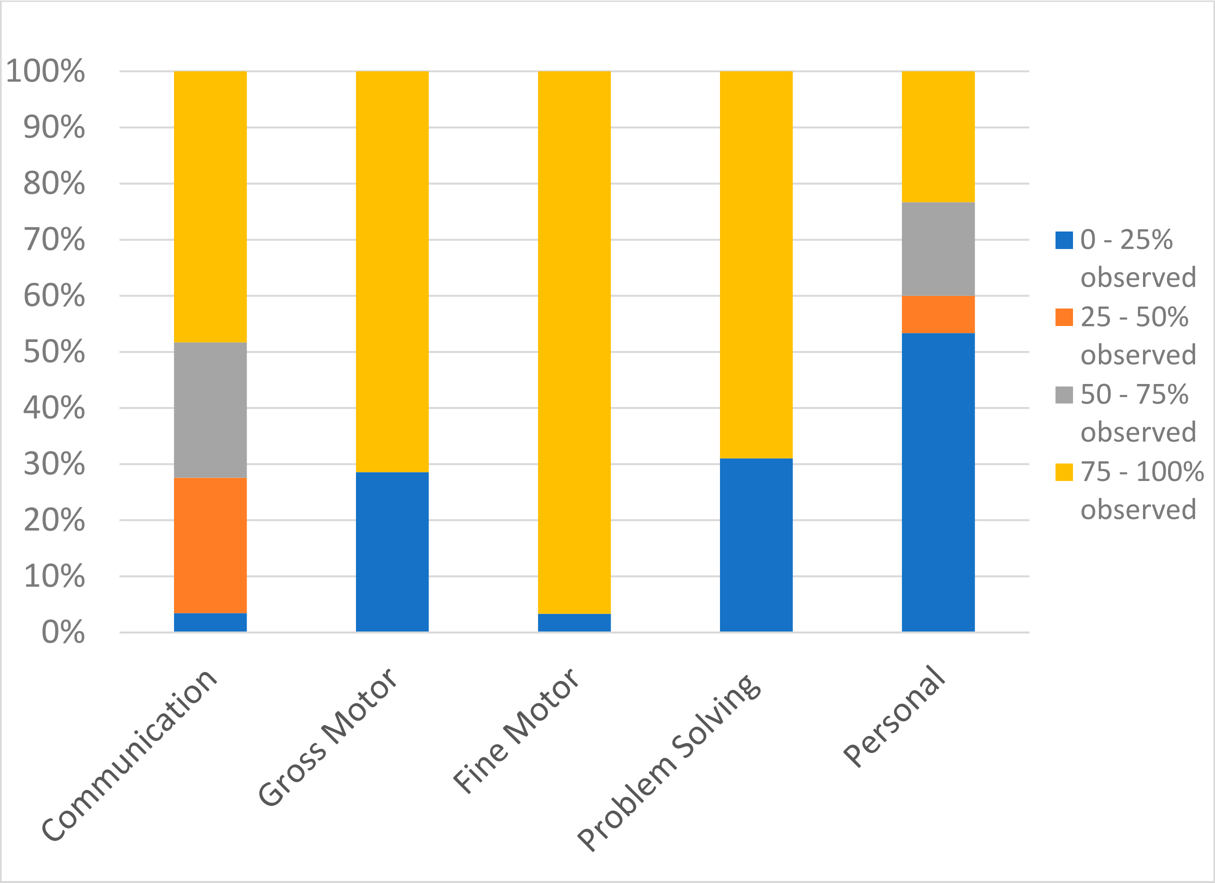

Supplementary Figure 1. Percentage of items in each subscale observed by examiners during sessions
